# Case Report: Incidental finding of an atresia of the inferior vena cava—a challenge for cardiac surgery

**DOI:** 10.3389/fcvm.2024.1321685

**Published:** 2024-02-06

**Authors:** Joscha Buech, Caroline Radner, Thomas Fabry, Simon Rutkowski, Christian Hagl, Sven Peterss, Maximilian A. Pichlmaier

**Affiliations:** ^1^ Department of Cardiac Surgery, LMU University Hospital, Munich, Germany; ^2^ German Centre for Cardiovascular Research (DZHK), Partner Site Munich Heart Alliance, Munich, Germany; ^3^ University Aortic Center Munich (LMU), LMU University Hospital, Munich, Germany

**Keywords:** aortic surgery, thoracic aorta aneurysm, anatomic variant, vena cava inferior agenesia, computer tomograph, preoperating planning

## Abstract

Inferior vena cava atresia is a rare and usually asymptomatic condition. However, when these patients undergo cardiac surgery, it can present an unexpected and challenging situation for the surgeon. Specifically, adequate venous drainage during cardiopulmonary bypass (CPB) is a critical issue here and may require an extension of cannulation strategies. Adequate preoperative diagnostics, ideally with imaging modalities such as CT angiography or MRI, are required for optimal surgical planning. Here, we describe a rare case of thoracic ascending aortic aneurysm with concomitant inferior vena cava atresia that was successfully operated on. With adequate preoperative planning, we were able to perform an operation without unforeseen complications with standard initialization of CPB.

## Introduction

Continuity of the azygos vein with an interrupted or totally absent inferior vena cava (IVC) is a rare congenital anomaly affecting 0.3% of otherwise healthy individuals (1). IVC interruption may be found in up to 2% of patients presenting with other cardiovascular defects such as dextrocardia, atrial septal defect, atrioventricular canal, and pulmonary artery stenosis (2). In everyday life, patients with an interruption of the IVC are often asymptomatic but have an increased risk of venous thrombosis (3, 4). If cardiac surgery with CPB is required, strategies are needed to ensure unobstructed venous drainage in these patients, as venous return may be compressed in the supine position on the operating table due to the lack of IVC. Therefore additional venous cannulation e.g., of the femoral vein may be required to allow the CPB to perform the a full calculated cardiac output. Furthermore, if unknown to the surgeon, agenesis of the IVC may lead to fatal injury to the hepatic veins during the attempt to cannulate the non-existent IVC via the right atrium.

Here, we describe the case of a patient with incidental finding of an agenesis of the IVC and continuity of the azygos vein who underwent aortic surgery.

## Case presentation

**Figure 1:**
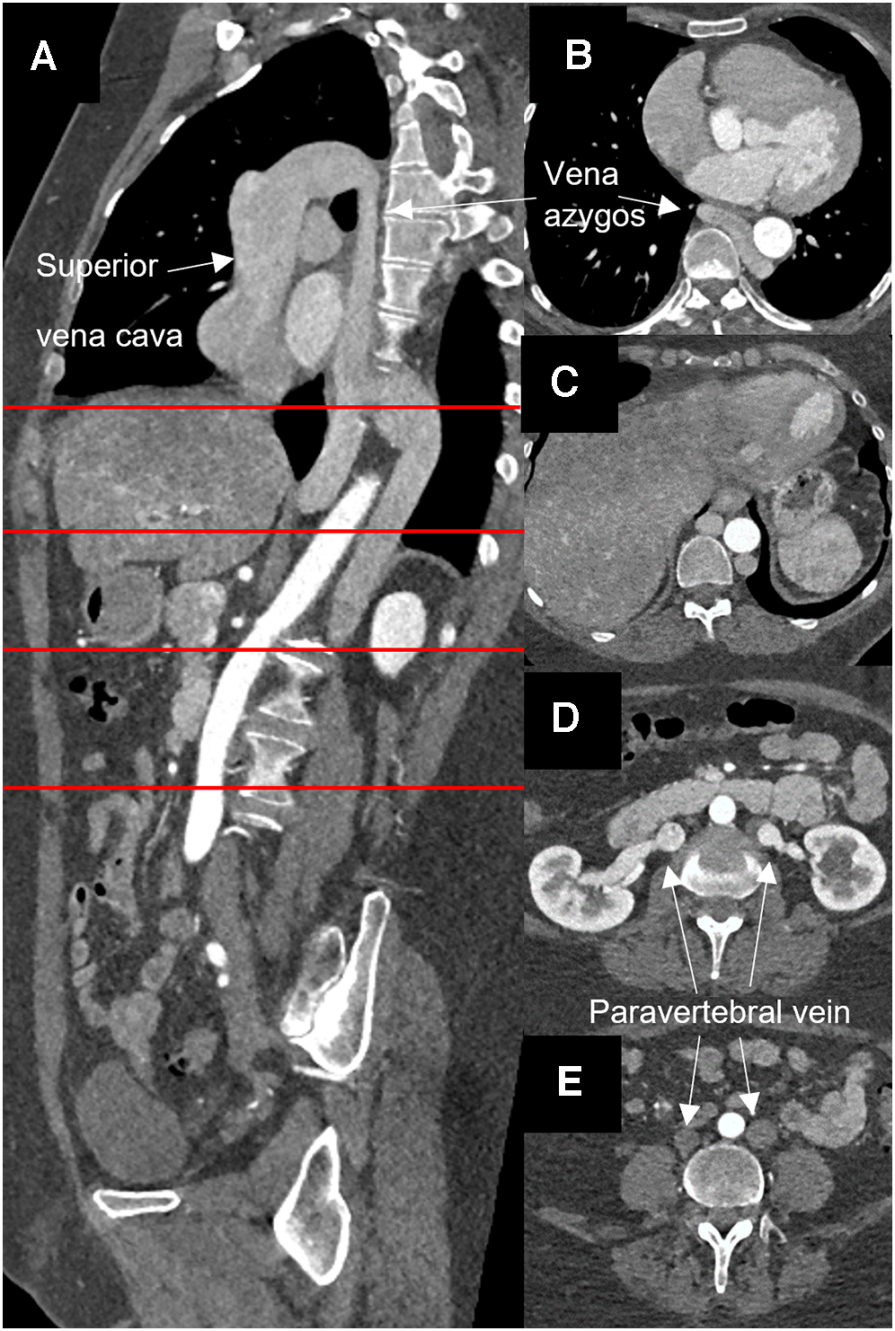
Computed angiopgraphy showing atresia of the inferior vena cava and azygos continuity. (**A**) Sagital layer, (**B–E**) horizontal layer at the sites marked in red.

A 54-year-old woman presented with severe aortic valve stenosis and an ascending aortic aneurysm. Echocardiography revealed a maximum gradient across the aortic valve of 58 mmHg, a calculated valve opening area of 0.7 cm^2^, a bicuspid valve morphology, and ventricular hypertrophy. Left ventricular function was normal. CT angiography confirmed an aneurysm of the ascending aorta of 46 × 45 mm. Additionally, an azygos continuity with complete agenesis of the inferior vena cava was found incidentally in routine CT angiography. Both iliac veins were found to directly drain into the corresponding paravertebral vein and then merged with the left and right renal veins to form a large azygos vein (Figure 1).

An elective replacement of the aortic root, ascending aorta and proximal aortic arch was scheduled. During induction of anaesthesia, an additional distal venous pressure measurement was established via a femoral vein catheter. Initial measurement showed no difference to the central venous pressure measured in the internal jugular vein. Intraoperatively, this monitoring strategy served to ensure adequate drainage of the lower body throughout CPB.

**Figure 2:**
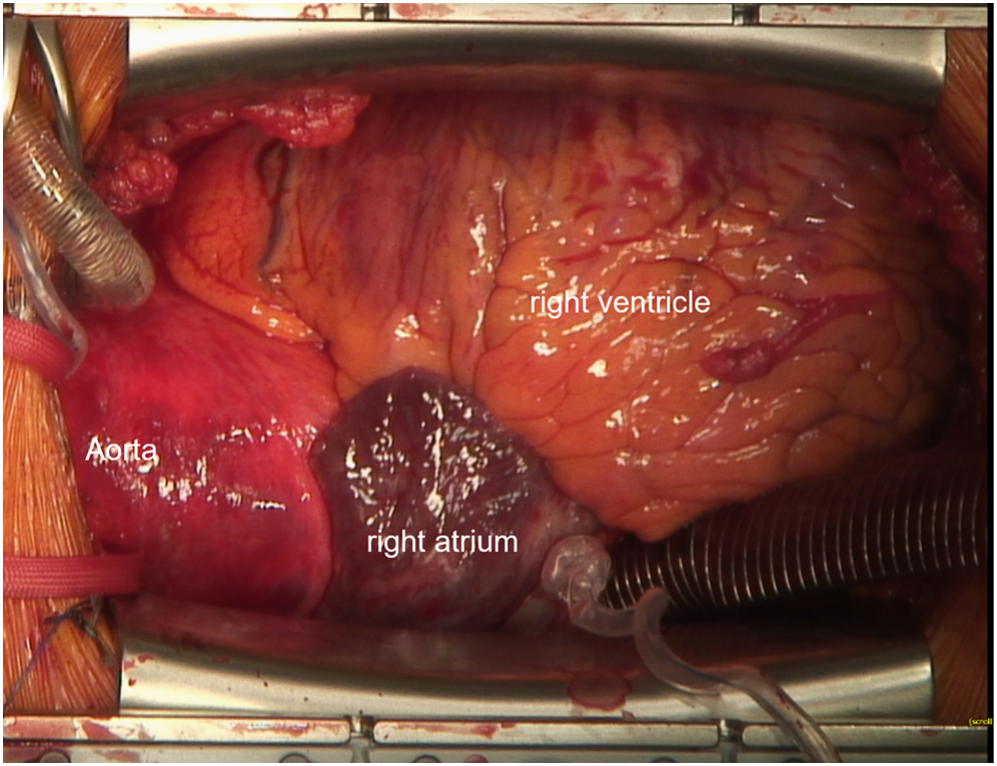
Intraoperative cannulation. Venous cannulation with a 2-stage cannula into the superior vena cava is shown.

Following median sternotomy and pericardiotomy, cannulation of the ascending aorta as well as the superior vena cava via the right atrium were performed, the latter using a regular two-stage cannula directed cranialwards (Figure 2). CPB was initiated and proved satisfactory considering venous drainage and full relief of the heart. Additional femoral venous cannulation was not required. For cardiac arrest, ice-cold (5–8°C) crystalloid solution was used. Additionally topical cooling with cold water was performed. The aortic valve, aortic root, and ascending aorta were replaced using a mechanical 23 mm conduit. The hemiarch was replaced with a 26 mm tube under hypothermic circulatory arrest (25 min) with selective antegrade cerebral perfusion (SACP). Therefore, after reaching a body temperature of 25°C, SACP was initiated by open direct cannulation of the innominate artery and the left common carotid artery. The left subclavian artery was blocked with a ballon catheter. The total duration of the cardiopulmonary bypass was 4 h and 26 min, and aortic clamp time was 2 h and 46 min.

The patient was transferred to the intensive care unit. Weaning from ventilation took place 12 h after surgery, and the patient was transferred to the intermediate care ward. During the hospital stay, the patient experienced a pericardial effusion requiring re- sternotomy. The remaining hospital stay was uneventful, and the patient was discharged in good physical condition. The predischarge CT angiography confirmed a satisfactory early postoperative result.

## Discussion

Atresia of the IVC with azygos continuity is a challenging condition in case cardiac surgery is needed. Recently, two cases of cardiac surgery with an aberrant IVC have been described. Cetinkaya et al. reported a case of a 61-year-old man with mitral and tricuspid valve replacement and an abnormal intraoperative IVC finding requiring conversion from a minimally invasive approach to a full sternotomy (5). Knol et al. describe a similar case of a 57-year-old man with mitral and tricuspid insufficiency where a full sternotomy was also required after initially starting with a minimally invasive approach for valve reconstruction (6). In both cases, additional cannulation of the femoral vein was required for adequate venous drainage. In this situation, a hybrid room for positioning the venous cannula with angiographic support could be useful as transesophageal echocardiography may not provide sufficient visibility to check the correct position. This should be considered in the preoperative planning. In our case, a central two-stage venous cannula directed upwards rather than downwards proved sufficient. Monitoring of the venous pressure in the iliac veins provided the critical information about sufficient venous drainage throughout the operation. This additional monitoring was also used for postoperative fluid management and catecholamine dosing in the intensive care unit.

This case underlines the importance of thorough preoperative diagnostics in elective cardiac surgery in order to be able to recognize and prepare different strategies even for such a rare anatomical variant. Non-invasive preoperative diagnostics such as computed tomography or magnetic resonance imaging becoming increasingly important in preoperative diagnostics and are suited to depict also the IVC but are not yet standard care in cardiac surgery (7, 8). The chest x-ray may show unspecific signs like a widened mediastinum with an enlarged azygos arch (9). In infants, ultrasound with the specific “double sign” may be an alternative (10). In adults, transesophageal ultrasound showing the interruption of back flow can be a diagnostic sign (11).

## Conclusion

We hereby describe a rare case of IVC atresia with azygos continuity in a patient undergoing thoracic aortic surgery. It could be demonstrated that, with proper preoperative diagnostics and planning, this challenging condition may even be addressed without the need for a strategy change during the operation.

## Data Availability

The original contributions presented in the study are included in the article/Supplementary Material, further inquiries can be directed to the corresponding author.
